# Investigating the significance of tumor-infiltrating immune cells for the prognosis of lung squamous cell carcinoma

**DOI:** 10.7717/peerj.7918

**Published:** 2019-10-25

**Authors:** Yueyan Zhu, Xiaoqin Zhang

**Affiliations:** Department of Respiratory Medicine, Zhejiang Provincial People’s Hospital, People’s Hospital of Hangzhou Medical College, Hangzhou, Zhejiang, China

**Keywords:** LSCC, TIICs, Prognosis, Immune risk score model, Nomogram

## Abstract

**Objective:**

Increasing evidence has indicated an association between immune cells infiltration in LSCC and clinical outcome. The aim of this research was tantamount to comprehensively investigate the effect of 22 tumor infiltrating immune cells (TIICs) on the prognosis of LSCC patients.

**Methods:**

In our research, the CIBERSORT algorithm was utilized to calculate the proportion of 22 TIICs in 502 cases from the TCGA cohort. Cases with a CIBERSORT *P*-value of <0.05 were kept for further study. Using the CIBERSORT algorithm, we first investigated the difference of immune infiltration between normal tissue and LSCC in 22 subpopulations of immune cells. Kaplan-Meier analysis was used to analyze the effect of 22 TIICs on the prognosis of LSCC. An immune risk score model was constructed based on TIICs correlated with LSCC-related recurrence. Multivariate cox regression analysis was used to investigate whether the immune risk score was an independent factor for prognosis prediction of LSCC. Nomogram was under construction to comprehensively predict the survival rate of LSCC.

**Results:**

The results of the different analysis showed that except of memory B cells, naive CD4+T cells, T cells and activated NK cells, the remaining immune cells all had differential infiltration in normal tissues and LSCC (*p* < 0.05). Kaplan-Meier analysis revealed two immune cells statistically related to LSCC-related recurrence, including activated mast cells and follicular helper T cells. Immune risk score model was constructed based on three immune cells including resting memory CD4+T cells, activated mast cells and follicular helper T cells retained by forward stepwise regression analysis. The Kaplan-Meier curve indicated that patients in the high-risk group linked to poor outcome (*P* = 8.277e−03). ROC curve indicated that the immune risk score model was reliable in predicting recurrence risk (AUC = 0.614). Multivariate cox regression analysis showed that the immune risk score model was just an independent factor for prognosis prediction of LSCC (HR = 2.99, 95% CI [1.65–5.40]; *P* = 0.0002). The nomogram model combined immune risk score and clinicopathologic parameter score to predict 3-year survival in patients with LSCC.

**Conclusions:**

Collectively, tumor-infiltrating immune cells play a major role in the prognosis of LSCC.

## Introduction

Lung cancer, as a common malignant tumor, is part of the leading causes of cancer-related death worldwide (Siegel, Miller & Jemal, 2017). Non-small cell lung cancer (NSCLC) is the most frequent subtype of lung cancer, LSCC as a histologic subtype of NSCLC accounts for more than 40% of the annual confirmed cases of lung cancer (Piperdi, Merla & Perez-Soler, 2014). Currently, the treatment and prognostic evaluation of LSCC mainly hinges on TNM stage, and surgical resection is the most suitable treatment for patients with early LSCC (Detterbeck, Boffa & Tanoue, 2009). However, although surgical resection improved survival, recurrence took place in nearly a quarter of patients (Baltayiannis et al., 2013; Fedor, Johnson & Singhal, 2013). Therefore, it is extremely important to accurately assess the recurrent risk in LSCC patients.

The infiltration of immune cells in the tumor is closely related to clinical consequences, and most likely to be used as drug targets to improve the survival rate of patients. Immunocheckpoint therapy is a type of therapy to improve the anti-tumor immune response by regulating T cell activity through co-inhibition or co-stimulation, which shows significant clinical effects (Davidson, Okines & Starling, 2015; Lote, Cafferkey & Chau, 2015; Newman et al., 2015; Schadendorf et al., 2015). With the development of immunocheckpoint therapy, the distribution of infiltrating immune cells in tumors has been the subject of research. Previous studies have primarily used flow cytometry or immunohistochemistry to assess the composition of infiltrating immune cells in tumors, but these methods have their limitations. Researchers recently developed a new bioinformatics tool called CIBERSORT (Charoentong et al., 2017). CIBERSORT, a deconvolution algorithm improved by Bindea et al. (2013), can estimate the cell composition of composite tissues based on standardized gene expression data. This method can quantify the abundance of specific cell types and has been properly validated by flow cytometry. The composition of immune cells in breast and liver cancer tissues has been successfully assessed by this method (Ali et al., 2016; Rohr-Udilova et al., 2018). Ali et al. (2016) showed that the difference of immune infiltrating cell composition in breast cancer may be an important factor in determining prognosis and treatment response. Rohr-Udilova et al. (2018) reported that monocytes, activated mast cells and plasma cells were decreased in HCC, while naïve B cells, resting mast cells, CD8+ T cells and CD4+ memory resting were increased when compared to healthy livers. In this study, gene expression data from 502 patients with LSCC based on the TCGA database were analyzed. CIBERSORT was used to assess the proportion of 22 immune cell types in tumor samples and to analyze their relationship with overall survival.

## Material and Methods

### Data acquisition

Training cohort of LSCC for this study were obtained from the shared database TCGA (The Cancer Genome Atlas) (Deng et al., 2016; Sato et al., 2013; Wang, Jensen & Zenklusen, 2016). We downloaded transcription data of 502 patients with LSCC from the TCGA database by typing the keyword “lung squamous cell carcinoma” of UCSC Xena website (https://xenabrowser.net/). These included 49 cases of normal lung tissue and 436 cases of LSCC. Secondly, we also obtained the quantifiable information including prognostic information, age, gender, stage, TNM stage and so on. Finally, we utilized the “lemma” package in R software to calibrate the transcription data of LSCC.

### Evaluation of tumor infiltrating immune cells

CIBERSORT is a deconvolution algorithm utilizing 547 labeled gene expression values to determine the proportion of 22 immune cells in tissues (Gentles et al., 2015; Newman et al., 2015). In this study, we used this algorithm to calculate the proportion of 22 infiltrating immune cells in LSCC tissues. We upload corrected transcription data to CIBERSORT website (http://cibersort.stanford.edu/). Each sample in the data set will get a *P* value, and samples with a *P* value less than 0.05 will be selected for further study.

### Statistical analyses

SPSS 23.0 (IBM, Armonk, NY, USA) and R 3.5.3 (R Core Team, 2019) were used for analysis. All statistical tests were bilateral, and a *P* value less than 0.05 was studied statistically significant. Continuous variables having to be in conformity with customary distribution were compared by independent *t* test, while continuous variables with skewed distribution were compared by Mann–Whitney *U* test. Pearson’s correlation analysis and spearman’s correlation analysis was employed in the correlation analysis. The Kaplan-Meier curve was utilized to analyze the relationship between immune risk score and overall survival. Log-rank test is employed to evaluation. Immune risk score model was constructed based on TIICs correlated with LSCC-related recurrence. Multivariate cox regression analysis was used to investigate whether the immune risk score was an independent factor for prognosis prediction of LSCC. The nomogram was under construction to comprehensively predict the survival rate of LSCC.

## Results

### The landscape of immune infiltration in LSCC

**Figure 1:**
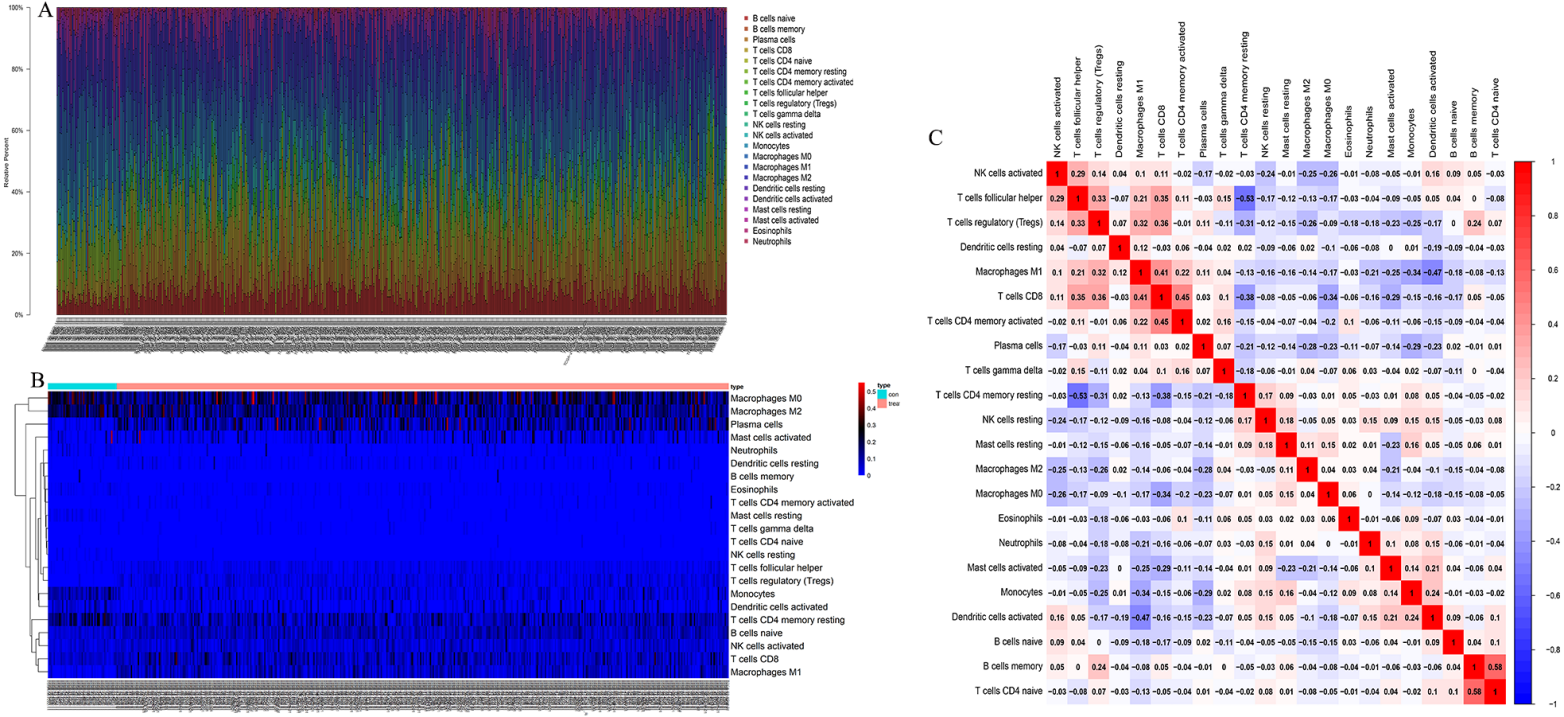
The landscape of Tumor-infiltrating immune cells in LSCC. (A) The proportion of 22 immune cells in LSCC tissues. (B) The heat maps of 22 immune cells in LSCC tissues, the horizontal axis shows the clustering information of samples which were divided into two major clusters. (C) Correlation matrix between 22 immune cells in LSCC, red means positive correlation, blue means negative correlation, and the darker the color, the stronger the correlation.

CIBERSORT algorithm was used to screen out samples with CIBERSORT output *P* value less than 0.05 for research, and 485 samples including 49 normal lung tissues and 436 LSCC tissues were screened out. We plotted bar plot to demonstrate the proportion of 22 immune cells in each sample (Fig. 1A). The results revealed that the five immune cells with the highest proportion in LSCC were M0 Macrophages (21.0%), M2 Macrophages (16.8%), Plasma cells (11.0%), resting memory CD4+ T cells (10%) and naive B cells (9.0%). Then, we plot the heat map of 22 immune cells in Fig. 1B. Figure 1C indicated the correlation coefficient between 22 immune cells, among which naive B cells and memory B cells have the strongest positive correlation (*r* = 0.58), resting memory CD4+ T cells had the strongest negative correlation with follicular helper T cells (*r* =  − 0.53).

### The different proportion of 22 immune cells in normal lung tissue and LSCC

**Figure 2:**
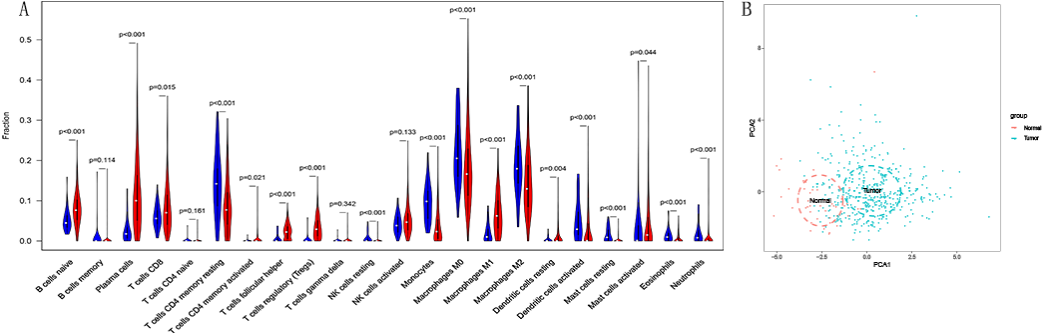
The different proportion of 22 immune cells in normal lung tissue and LSCC tissue. (A) Variance analysis, blue represents normal lung tissue, and red represents LSCC tissue. (B) Principal component analysis, the first two principal components which explain the most of the data variation are shown.

We compared the differential infiltration of 22 immune cells between normal lung tissues and LSCC tissues. The results showed that except for memory B cells, naive CD4+T cells, gamma delta T cells and activated NK cells, the remaining immune cells were infiltrated differently in normal lung tissues and LSCC tissues (*p* < 0.05, Fig. 2A). The results of principal component analysis showed that there are significant individual differences between normal lung tissues and LSCC tissues (*p* < 0.05, Fig. 2B).

### Predictive value of TIICs in LSCC

**Figure 3:**
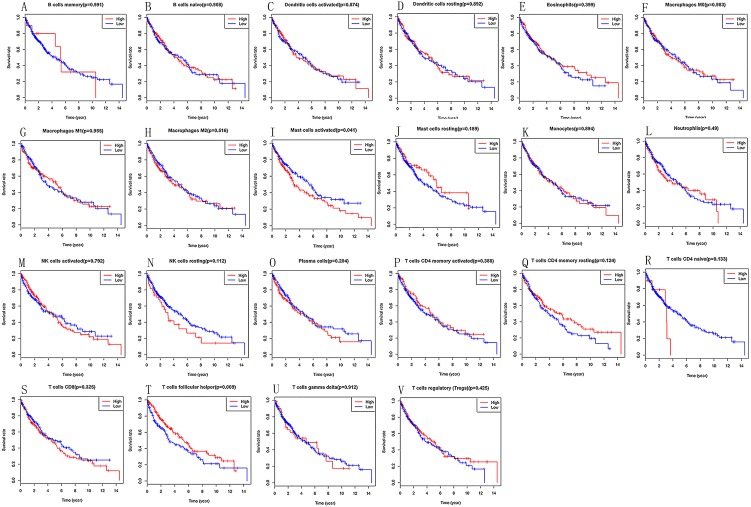
(A–V) Prognostic value of 22 immune cells in LSCC by Kaplan–Meier analysis, high infiltration of activated mast cells and follicular helper T cells in LSCC tissues are linked to poor prognosis.

Kaplan–Meier analysis was utilized to investigate the prognostic value of 22 tumours infiltrating immune cells in LSCC tissues. We can find that high infiltration of activated mast cells (*P* = 0.041) and follicular helper T cells (*P* = 0.009) in LSCC tissues are linked to poor prognosis (Fig. 3B).

### Establishment of immune risk score model

**Figure 4:**
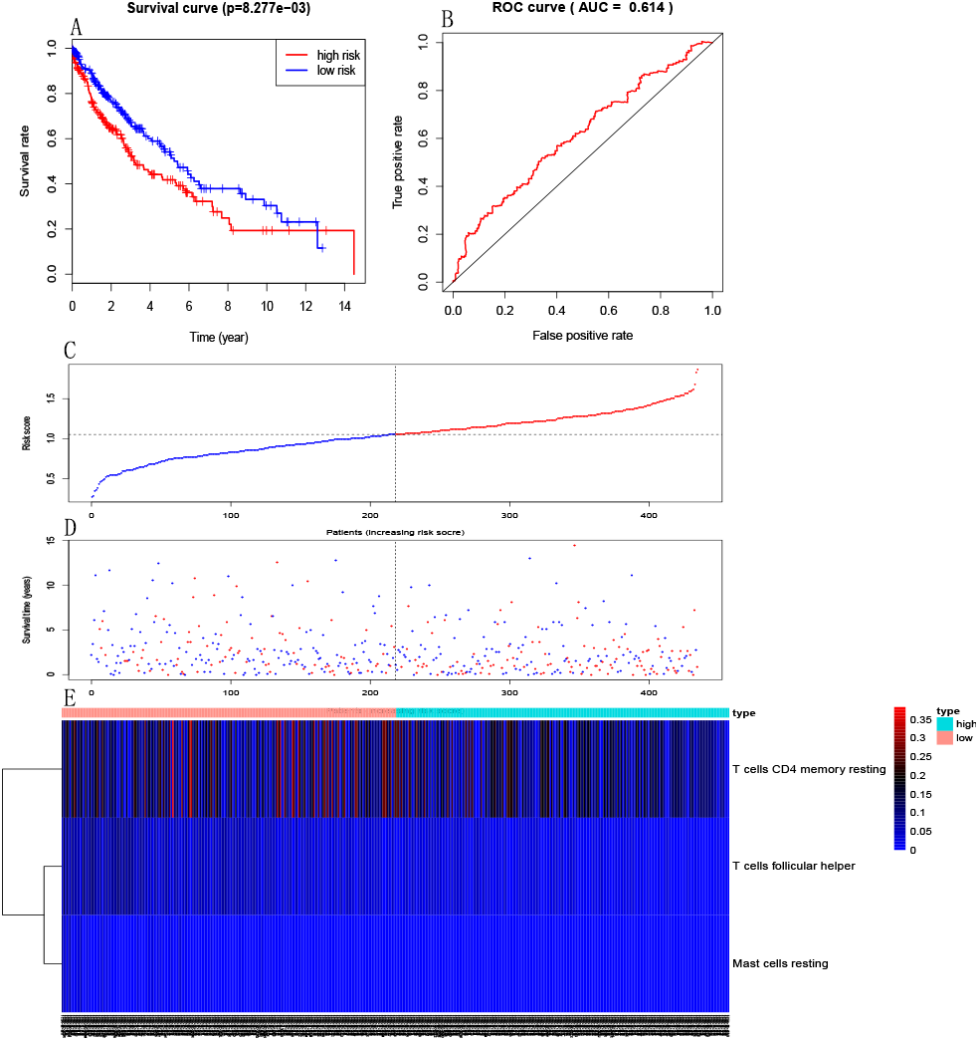
Establishment of immune risk scores model. (A) Kaplan–Meier survival curves of overall survival between high-risk and low-risk patients. (B) ROC curve AUC statistics assess the predictive power of the immune risk score model. (C) The distribution of patients’ risk score. (D) The distribution of patients’ survival state. (E) Three immune cell infiltration of patients with LSCC.

Multivariate Cox PHR was carried out to construct an excepted risk score model based on resting memory CD4+T cells, activated mast cells and follicular helper T cells selected by forward stepwise regression analysis. Formula is this: Risk3 = −3.03 * resting memory CD4 + T cells −15.26 * activated mast cells −21.67 * follicular helper T cells (Table 1). Each sample will be paid a risk score built on the model. Patients were divided into a high-risk group and a low-risk group according to the median risk score. Kaplan–Meier curves indicated that patients in the high-risk group had a poorer prognosis than those in the low-risk group (*p* = 8.277e−03, Fig. 4A). The ROC curve showed that the immune risk score model is reliable to predict the prognosis of patients with LSCC (AUC=0.614, Fig. 4B). In addition, Figures 4C, 4D and 4E respectively showed the risk score, survival status and three immune cells infiltration of patients with LSCC.

**Table 1:** Multivariate Cox PHR based on 3 immune cells.

id	Coef	HR	HR.95L	HR.95H	*p* value
T cells CD4 memory resting	−3.03219	0.04821	0.002677	0.868148	0.039799
T cells follicular helper	−15.2555	2.37E−07	2.34E−11	0.002394	0.001184
Mast cells resting	−21.6671	3.89E−10	3.29E−22	459.8077	0.126588

**Notes.**

Abbreviations HR hazard ratio

### Independent predictive power of immune risk score model

Multivariate analysis was used to investigate whether the risk score as predictors of overall survival was independent of other clinicopathological data such as age, gender and clinical stage. The results suggested that the risk score (HR = 1.30, 95% CI [1.20–1.40]; *P* < 0.001) and clinical stage (HR = 1.70, 95% CI [1.49–2.10]; *P* < 0.001) are two independent predictors of overall survival in LSCC patients (Table 2).

**Table 2:** Independent predictive power of immune risk score model.

id	Univariate analysis				Multivariate analysis			
	HR	HR.95L	HR.95H	*p* value	HR	HR.95L	HR.95H	*p* value
RiskScore	2.78	1.63	4.74	0.0002	2.99	1.65	5.4	0.0003
Sex	0.8	0.56	1.12	0.19	0.67	0.46	0.99	0.048
T	1.26	1.05	1.52	0.014	0.98	0.71	1.36	0.9
N	1.16	0.94	1.43	0.16	0.97	0.64	1.46	0.88
M	3.03	1.11	8.21	0.03	1.08	0.27	4.34	0.92
Stage	1.24	1.04	1.49	0.02	1.31	0.83	2.06	0.25
Age	1.02	1	1.04	0.01	1.03	1.01	1.06	0.003

**Notes.**

Abbreviations HR hazard ratio

### Correlation between immune risk score and clinicopathological parameters

To analyze the correlation between immune risk score (IRS) and clinicopathological parameters in 436 LSCC samples. The results revealed that immune risk score is associated with T stage of LSCC, while there was no correlation between the patient’s immune risk score and clinicopathological parameters such as age, gender, clinical stage, N stage and M stage (*P* > 0.05, Table 3).

**Table 3:** Correlation between the immune risk score and clinicopathological parameters.

Item		Mean IRS value	*P*
Age	<68	1.07	0.34
	>68	1.01	
Stage	I	1.05	0.9
	II	1.08	
	III	1.03	
	IV	1.09	
Gender	Female	1.05	0.96
	Male	1.05	
T	T1	1	0.03
	T2	1.07	
	T3	1.08	
	T4	0.94	
N	N0	1.05	0.69
	N1	1.06	
	N2	1.04	
	N3	1.15	
M	M0	1.06	0.98
	M1	1.09	

**Notes.**

Abbreviations IRS immune risk score

### Construction of nomogram model

**Figure 5:**
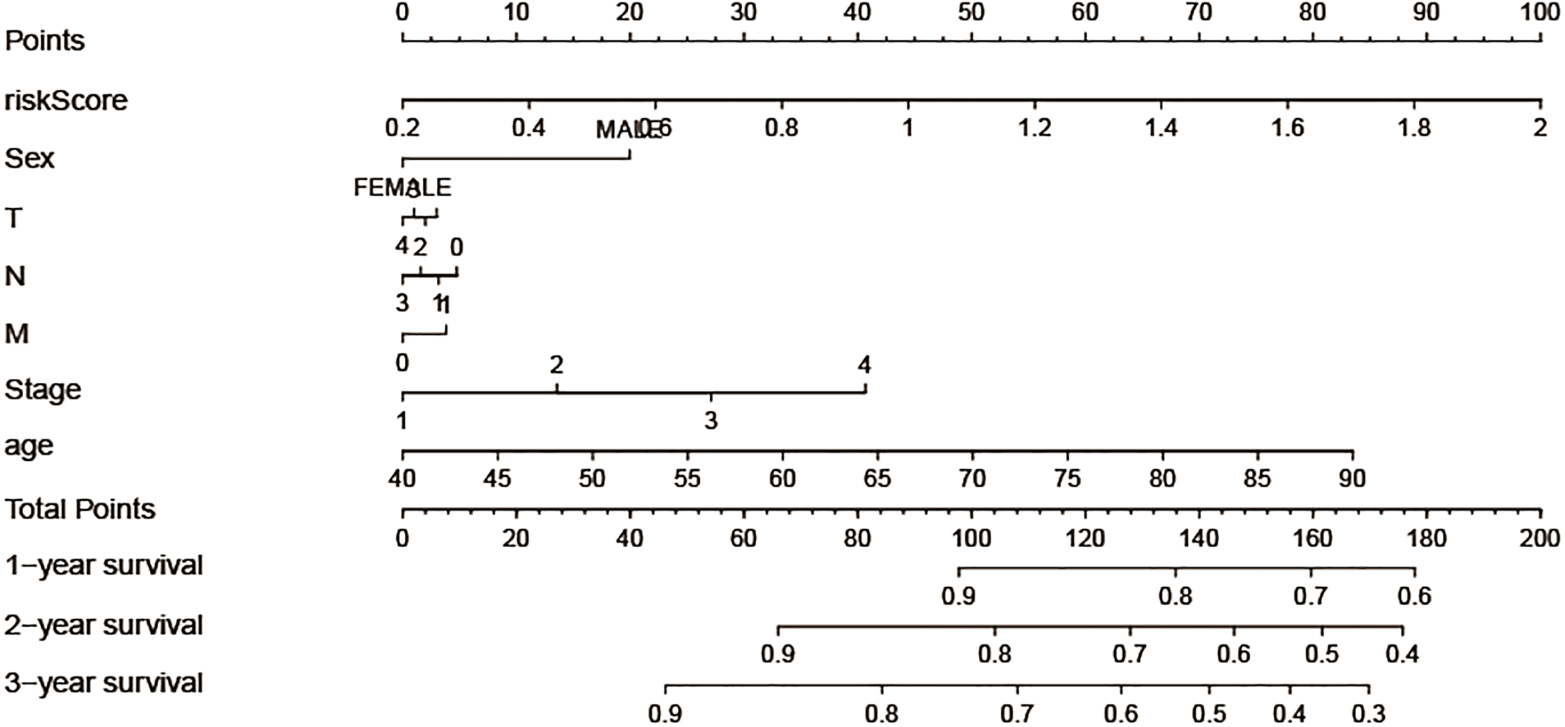
Construction of the nomogram model, each variable axis represented an individual risk factor, and the line drawn upwards was used to determine the points of each variable. Then the total points would be calculated to obtain the probability of 1-, 2- and 3-year OS.

In order to take full advantage of the clinicopathological parameters of LSCC and the excepted risk model to predict the survival rates of LSCC patients, we constructed a nomogram. We can take note of the prognosis of patients according to clinicopathological parameters and immune risk model. Then, total scores were used to evaluate 3-year survival in patients with LSCC (Fig. 5).

## Discussion

Built on the “seed and soil” theory of cancer metastasis proposed by Paget, cancer cells, as “seed”, depend on the surrounding microenvironment “soil” for their occurrence and metastasis (Paget, 1989). Tumor microenvironment (TME) relates to bioactive molecules secreted by extra-cellular matrix (ECM), stomatal cells, tumor and stomatal cells, as well as lymphatic and vascular systems (Hanahan & Weinberg, 2011). On the one hand, tumor cells affect the surrounding microenvironment by autocrine and paracrine to continue their invasion, growth and tumor formation. On the other hand, various cells and extracellular matrix in tumor microenvironment play an important role in tumor development, invasion, metastasis and tumor treatment. Nowadays, a series of studies have shown that tumor immune cells as an important component of tumor microenvironment play a major role in tumor prognosis. For example, Liu et al. (2019) stated that Tregs T cells and M2 macrophages have different prognostic values in gastric cancer patients with distinct clinicopathological characteristics and chemotherapy strategies. Elevated tumor infiltrating lymphocytes were significantly related to better survival in colorectal cancer (Ko & Pyo, 2019).

At present, the conventional surgical method utilised for the treatment of LSCC is still resection. Being dependent on the investigation data, there is still a high recurrence rate after the resection of LSCC (Wang et al., 2019). Therefore, it is of profound significance to study the factors affecting the prognosis of LSCC patients to increase their long-term survival. To explore the prognostic value of tumor infiltrating immune cells in LSCC, CIBERSORT algorithm was used to calculate the proportion of 22 infiltrating immune cells in LSCC tissues, and samples with *P* value less than 0.05 were selected for this study. Kaplan–Meier analysis of 22 immune cells showed that activated mast cells were linked to poor prognosis of LSCC, while follicular helper T cells were associated with a better outcome of LSCC. Mast cells, as an important component of tumor microenvironment, have been proved to exist in a large number of solid tumors (Oldford & Marshall, 2015; Ribatti, 2016). Mast cells play both positive and negative roles in tumors, depending on bioactive substances secreted (Ribatti, 2016). A large number of studies have shown that high infiltration mast cells in tumors are associated with a good prognosis of patients (Carlini et al., 2010; Dabiri et al., 2004; Welsh et al., 2005), which runs counter to our results. Follicular helper T cells induce B cells to begin antibody responses outside the follicle and the germinal center. Previous studies have shown that invasive follicular helper T cells have a protective effect in colorectal cancer and breast cancer, which are substantially corelated with patient survival (Zhang et al., 2019). A multivariate cox regression model was used to construct the immune risk score model based on resting memory CD4+T cells, activated mast cells and follicular helper T cells selected by forward stepwise regression analysis, and the ROC curve indicated that the model was reliable in predicting the recurrence risk of LSCC. In addition, we tried to look for datasets in the GEO database to validate our results, but due to the limited number of LSCC patients, we were unable to make meaningful validation results. Given the rapid development of high-throughput technologies, it is reasonable to suppose that our immune risk score model has great potential for transforming clinical practice. In addition, we also found that naive B cells, memory B cells, plasma cells, CD8+T cells, memory CD4+T cells, trees T cells, resting NK cells, mast cells, monocytes cells and other cells had no statistical significance on the prognosis of LSCC. However, these cells show differential expression in normal lung tissues and LSCC tissues, suggesting that they are closely connected with the occurrence and progress of LSCC. Besides, correlation analysis showed that immune risk score is associated with T stage of LSCC, while there was no correlation between the patient’s immune risk score and clinicopathological parameters such as age, gender, clinical stage, N stage and M stage. The result indicated that the immune risk score is associated with local infiltration of LSCC, but not with distant metastasis. Finally, a nomogram model was constructed to predict the survival rates of LSCC patients. The line segment length corresponding to each variable in the nomogram represents the contribution of predictors to survival outcome. The immune risk score has the greatest effect on prognosis, while T stage has a smaller effect on prognosis compared with other factors. In future studies, external data should be used to verify the wide applicability of the nomogram.

## Conclusion

In conclusion, the present study demonstrated the prognostic value of 22 immune cells in LSCC. The immune risk score model was reliable for predicting the prognosis of LSCC based on the TCGA database, and the risk score model was an independent factor affecting the prognosis of LSCC. However, due to the limited number of LSCC patients, this immune risk score model could not be verified in GEO database. It is hoped that sufficient samples can be collected in future studies to verify this result.

##  Supplemental Information

10.7717/peerj.7918/supp-1 Supplemental Information 1 Clinical data

## Additional Information and Declarations

### Data Availability

The following information was supplied regarding data availability: This work uses public datasets. Raw data is available at GEO (GSE37745) and from the TCGA database (TCGA-LSCC).

## References

[ref-1] Ali HR, Chlon L, Pharoah PD, Markowetz F, Caldas C (2016). Patterns of immune infiltration in breast cancer and their clinical implications: a gene-expression-based retrospective study. PLOS Medicine.

[ref-2] Baltayiannis N, Chandrinos M, Anagnostopoulos D, Zarogoulidis P, Tsakiridis K, Mpakas A, Machairiotis N, Katsikogiannis N, Kougioumtzi I, Courcoutsakis N, Zarogoulidis K (2013). Lung cancer surgery: an up to date. Journal of Thoracic Disease.

[ref-3] Bindea G, Mlecnik B, Tosolini M, Kirilovsky A, Waldner M, Obenauf AC, Angell H, Fredriksen T, Lafontaine L, Berger A, Bruneval P, Fridman WH, Becker C, Pages F, Speicher MR, Trajanoski Z, Galon J (2013). Spatiotemporal dynamics of intratumoral immune cells reveal the immune landscape in human cancer. Immunity.

[ref-4] Carlini MJ, Dalurzo MC, Lastiri JM, Smith DE, Vasallo BC, Puricelli LI, Lauria de Cidre LS (2010). Mast cell phenotypes and microvessels in non-small cell lung cancer and its prognostic significance. Human Pathology.

[ref-5] Charoentong P, Finotello F, Angelova M, Mayer C, Efremova M, Rieder D, Hackl H, Trajanoski Z (2017). Pan-cancer immunogenomic analyses reveal genotype-immunophenotype relationships and predictors of response to checkpoint blockade. Cell Reports.

[ref-6] Dabiri S, Huntsman D, Makretsov N, Cheang M, Gilks B, Bajdik C, Gelmon K, Chia S, Hayes M (2004). The presence of stromal mast cells identifies a subset of invasive breast cancers with a favorable prognosis. Modern Pathology.

[ref-7] Davidson M, Okines AF, Starling N (2015). Current and future therapies for advanced gastric cancer. Clinical Colorectal Cancer.

[ref-8] Deng M, Bragelmann J, Schultze JL, Perner S (2016). Web-TCGA: an online platform for integrated analysis of molecular cancer data sets. BMC Bioinformatics.

[ref-9] Detterbeck FC, Boffa DJ, Tanoue LT (2009). The new lung cancer staging system. Chest.

[ref-10] Fedor D, Johnson WR, Singhal S (2013). Local recurrence following lung cancer surgery: incidence, risk factors, and outcomes. Surgical Oncology.

[ref-11] Gentles AJ, Newman AM, Liu CL, Bratman SV, Feng W, Kim D, Nair VS, Xu Y, Khuong A, Hoang CD, Diehn M, West RB, Plevritis SK, Alizadeh AA (2015). The prognostic landscape of genes and infiltrating immune cells across human cancers. Nature Medicine.

[ref-12] Hanahan D, Weinberg RA (2011). Hallmarks of cancer: the next generation. Cell.

[ref-13] Ko YS, Pyo JS (2019). Clinicopathological significance and prognostic role of tumor-infiltrating lymphocytes in colorectal cancer. International Journal of Biological Markers.

[ref-14] Liu X, Xu D, Huang C, Guo Y, Wang S, Zhu C, Xu J, Zhang Z, Shen Y, Zhao W, Zhao G (2019). Regulatory T cells and M2 macrophages present diverse prognostic value in gastric cancer patients with different clinicopathologic characteristics and chemotherapy strategies. Journal of Translational Medicine.

[ref-15] Lote H, Cafferkey C, Chau I (2015). PD-1 and PD-L1 blockade in gastrointestinal malignancies. Cancer Treatment Reviews.

[ref-16] Newman AM, Liu CL, Green MR, Gentles AJ, Feng W, Xu Y, Hoang CD, Diehn M, Alizadeh AA (2015). Robust enumeration of cell subsets from tissue expression profiles. Nature Methods.

[ref-17] Oldford SA, Marshall JS (2015). Mast cells as targets for immunotherapy of solid tumors. Molecular Immunology.

[ref-18] Paget S (1989). The distribution of secondary growths in cancer of the breast. Cancer and Metastasis Reviews.

[ref-19] Piperdi B, Merla A, Perez-Soler R (2014). Targeting angiogenesis in squamous non-small cell lung cancer. Drugs.

[ref-20] R Core Team (2019). R: a language and environment for statistical computing.

[ref-21] Ribatti D (2016). Mast cells in lymphomas. Critical Reviews in Oncology/Hematology.

[ref-22] Rohr-Udilova N, Klinglmuller F, Schulte-Hermann R, Stift J, Herac M, Salzmann M, Finotello F, Timelthaler G, Oberhuber G, Pinter M, Reiberger T, Jensen-Jarolim E, Eferl R, Trauner M (2018). Deviations of the immune cell landscape between healthy liver and hepatocellular carcinoma. Scientific Reports.

[ref-23] Sato Y, Yoshizato T, Shiraishi Y, Maekawa S, Okuno Y, Kamura T, Shimamura T, Sato-Otsubo A, Nagae G, Suzuki H, Nagata Y, Yoshida K, Kon A, Suzuki Y, Chiba K, Tanaka H, Niida A, Fujimoto A, Tsunoda T, Morikawa T, Maeda D, Kume H, Sugano S, Fukayama M, Aburatani H, Sanada M, Miyano S, Homma Y, Ogawa S (2013). Integrated molecular analysis of clear-cell renal cell carcinoma. Nature Genetics.

[ref-24] Schadendorf D, Hodi FS, Robert C, Weber JS, Margolin K, Hamid O, Patt D, Chen TT, Berman DM, Wolchok JD (2015). Pooled analysis of long-term survival data from phase ii and phase iii trials of ipilimumab in unresectable or metastatic melanoma. Journal of Clinical Oncology.

[ref-25] Siegel RL, Miller KD, Jemal A (2017). Cancer statistics, 2017. A Cancer Journal for Clinicians.

[ref-26] Wang Z, Jensen MA, Zenklusen JC (2016). A practical guide to the cancer genome Atlas (TCGA). Methods in Molecular Biology.

[ref-27] Wang W, Men Y, Wang J, Zhou Z, Chen D, Xiao Z, Feng Q, Lv J, Liang J, Bi N, Gao S, Wang L, Hui Z (2019). Postoperative radiotherapy is effective in improving survival of patients with stage pIII-N2 non-small-cell lung Cancer after pneumonectomy. BMC Cancer.

[ref-28] Welsh TJ, Green RH, Richardson D, Waller DA, O’Byrne KJ, Bradding P (2005). Macrophage and mast-cell invasion of tumor cell islets confers a marked survival advantage in non-small-cell lung cancer. Journal of Clinical Oncology.

[ref-29] Zhang R, Qi CF, Hu Y, Shan Y, Hsieh YP, Xu F, Lu G, Dai J, Gupta M, Cui M, Peng L, Yang J, Xue Q, Chen-Liang R, Chen K, Zhang Y, Fung-Leung WP, Mora JR, Li L, Morse 3rd HC, Ozato K, Heeger PS, Xiong H (2019). T follicular helper cells restricted by IRF8 contribute to T cell-mediated inflammation. Journal of Autoimmunity.

